# Efficacy of Combining Conventional Physiotherapy for Migraine With Transcutaneous Stimulation of the Auricular Vagal Nerve or Supraorbital Nerve: Protocol for a Randomized Controlled Trial

**DOI:** 10.2196/90669

**Published:** 2026-09-15

**Authors:** Nikita Deshmukh, Soumik Basu

**Affiliations:** 1 Department of Musculoskeletal Physiotherapy Ravi Nair Physiotherapy College Datta Meghe Institute of Higher education and Research Center Wardha, Maharashtra India

**Keywords:** migraine, auricular vagal nerve stimulation, supraorbital nerve stimulation, migraine without aura, neck pain

## Abstract

**Background:**

Migraine is a common and disabling neurological disorder characterized by recurrent headaches and associated symptoms that significantly affect quality of life. Conventional physiotherapy plays a supportive role in migraine management; however, it may not adequately address central sensitization and altered pain modulation. Noninvasive neuromodulation techniques, such as transcutaneous auricular vagal nerve stimulation (ta-VNS) and transcutaneous supraorbital nerve stimulation (t-SNS), have shown potential in modulating central pain pathways and reducing migraine burden.

**Objective:**

The primary objective of this study is to evaluate and compare the effectiveness of ta-VNS and t-SNS, each combined with conventional physiotherapy, in reducing pain intensity and migraine frequency in individuals with migraine. Secondary objectives include assessing their effects on migraine disability, neck disability, and cervical range of motion.

**Methods:**

This randomized controlled trial will include individuals clinically diagnosed with migraine. Participants will be randomly allocated into 2 groups: group A will receive ta-VNS along with conventional physiotherapy, and group B will receive t-SNS along with conventional physiotherapy. Both interventions will be administered for a defined treatment period. Outcome measures will be recorded at baseline, immediately after the intervention, and during follow-up periods to evaluate short- and long-term effects.

**Results:**

It is anticipated that both ta-VNS and t-SNS, when combined with conventional physiotherapy, will lead to significant improvements in pain intensity, migraine frequency, and functional outcomes. One neuromodulation technique may demonstrate greater or more sustained benefits than the other.

**Conclusions:**

This study is expected to provide comparative evidence on the effectiveness of ta-VNS and t-SNS as adjuncts to conventional physiotherapy in migraine management. The findings may support the inclusion of targeted noninvasive neuromodulation techniques in physiotherapy-based treatment protocols for migraine.

**Trial Registration:**

Clinical Trials Registry–India CTRI/2026/01/100045; https://ctri.nic.in/Clinicaltrials/pmaindet2.php?EncHid=MTQ3Mjgz&Enc=&userName=

**International Registered Report Identifier (IRRID):**

DERR1-10.2196/90669

## Introduction

Migraines are often associated with numerous symptoms of autonomic nervous system dysfunction, and they are characterized by recurrent moderate to severe headaches. The word “migraine” is derived from the Greek word ἡμικρανία (*hemikrania* or “pain on one side of the head” from ἡμι- [*hemi-* or “half”] and κρανίον [*kranion* or “skull”]) [1]. According to the *International Classification of Diseases, 9th Revision* (ICD-9) and *International Classification of Diseases, 10th Revision* (ICD-10), it is classified under codes 346-346.93 and G43-G43.919 [2]. In the *International Classification of Headache Disorders–3* (ICHD-3) classification, migraine is divided into three main types [3].

The first is migraine type is without aura, typically a unilateral, pulsatile, and moderate to severe headache lasting from 4 to 72 hours. The headache often worsens in intensity with increased physical activity and is associated with nausea, vomiting, phonophobia, and photophobia. Prodromal symptoms may include fatigue, mood changes, and food cravings; postdromal symptoms such as tiredness and neck stiffness may follow.

The second type is migraine with aura, which involves at least 2 attacks with fully reversible symptoms such as visual, sensory, speech, motor, brainstem, or retinal disturbances. Features include gradual symptom onset over ≥5 minutes, sequential symptoms, a duration of 5 to 60 minutes, and the possibility of a headache within 60 minutes.

The third type is chronic migraine, in which the patient experiences headaches for >15 days per month, with migraine features, with or without aura on ≥8 days per month for ≥3 months. The symptoms may occur either with or without aura [3].

Migraine is usually an inherited disorder, and the origin of an attack is related to neuronal activation. The site of origin of attacks remains controversial. Two current concepts of migraine origin are cortical spreading depression (CSD) and a brainstem generator. CSD is the basis of migraine aura. It consists of cortical neuronal activation and a postictal depression of neuronal firing. CSD may trigger meningeal pain mechanisms through neurogenic inflammation, vasodilatation, and plasma protein extravasation. CSD further disrupts the blood-brain barrier by activating brain matrix metalloproteinases, thereby opening the blood-brain barrier and potentially leading to migraine pain. CSD is also associated with the activation of cortical glutamatergic synapses. Following the onset of migraine by CSD or a brainstem generator, meningeal pain mechanisms are initiated via trigeminovascular activation. Vasodilatation and neurogenic inflammation sensitize the trigeminovascular sensory fibers that carry pain signals via the trigeminal ganglion to the trigeminal nucleus caudalis. Activation of these peripheral nociceptors is called peripheral sensitization. Activation of the trigeminal nucleus caudalis and rostral brain structures is termed central sensitization [4]. Neck muscle stiffness is often associated with repeated migraine attacks, which may lead to central sensitization and lower the pain pressure threshold. Neck muscle stiffness could be caused directly by alterations in the muscles, such as inflammation or trigger points, which may activate sensory neurons and thereby contribute to migraine pain [5]. The global prevalence of migraine is estimated to be 15% to 18%, with chronic migraine affecting approximately 1% to 2% of the general population [6]. Approximately 15% to one-third (approximately 25%-33%) of people with migraine experience aura [7]. Migraine without aura is more common, affecting approximately 63.9% of patients with migraine in population-based studies [8]. The prevalence of migraine is 26.3% in India [9] and 30% in Maharashtra [10]. Neck pain is 2 times higher in patients with chronic migraine than in patients with episodic migraine. At the onset of a migraine attack, 37.8% of patients experience neck pain, and within the 2 hours before the onset of a migraine attack, 24.2% of patients experience neck pain (in 82.2% of these patients, neck pain continued into the attack state), and 7.4% experienced neck pain from 2 to 48 hours before the onset of an attack. A comparable neck pain prevalence of 76.2% was reported in the only population-based study of adult patients with migraine [11].

Migraine without aura is a prevalent and highly disabling neurological condition. According to the ICHD-3, chronic migraine is characterized by headache occurring on >15 days per month for >3 months, with migraine features present on ≥8 days per month. Approximately 1.4% to 2.2% of the global population is affected by chronic migraine. According to the World Health Organization (WHO) and the Global Burden of Disease (GBD) Study 2019, migraine is the second leading cause of years lived with disability (YLDs) worldwide and the leading neurological disorder in terms of disability [12]. Migraine is often accompanied by neck pain, which contributes to increased disability and complicates management [5]. The reviewed literature demonstrates diverse physiotherapy approaches to migraine management, including pharmacological and nonpharmacological treatment. Although many of these interventions, such as transcutaneous auricular vagal nerve stimulation (ta-VNS), transcutaneous supraorbital nerve stimulation (t-SNS), and conventional physiotherapy, have shown promising results in alleviating migraine symptoms, there remains a significant gap in studies focusing on outcomes specifically in chronic migraine.

Moreover, none of the studies have directly compared the efficacy of ta-VNS and t-SNS, creating a comparative evidence gap. This study will determine whether these neuromodulatory mechanisms may provide an effective treatment for migraine. Such research could contribute to a more integrated and personalized care model for patients with chronic migraine, optimizing therapeutic outcomes and guiding clinical decision-making.

## Methods

### Objective and Trial Design

This randomized controlled noninferiority trial will compare effects of ta-VNS and t-SNS in patients with chronic migraine. Participants will be randomly assigned in a 1:1 ratio to either ta-VNS or t-SNS. Outcome assessors will, however, be blinded to group allocation.

### Recruitment and Study Setting

After approval by the ethics committee (DMIHER[DU]/IEC/2025/562), eligible participants according to the inclusion criteria will be recruited at the physiotherapy outpatient department (OPD), Centre of Neurosciences and Medicine, Acharya Vinoba Bhave Rural Hospital, Datta Meghe Institute of Higher Education and Research (DMIHER; Deemed to be University [DU]), Sawangi (Meghe), Wardha. All participants will be required to provide written informed consent before participating in the study. Participants selected according to the inclusion criteria will be randomly assigned in a 1:1 ratio to 2 groups. Before the initiation of treatment, the preintervention outcome measures will be recorded, followed by assessments at baseline and at 4, 8, and 12 weeks.

### Participant Eligibility Criteria

The inclusion and exclusion criteria are provided in Textbox 1.

**Eligibility criteria for study participation.**

**Inclusion criteria**

- Participants who provide written informed consent
- Both male and female participants, aged 18 to 40 years
- Chronic migraine diagnosed by a neurologist
- A history of migraine lasting >3 months
- Use of prophylactic medication for migraine during the past 3 months
- Moderate to severe headache occurring 15 days per month, including 8 migraine days, with a pulsatile and unilateral quality and accompanied by nausea, vomiting, and intolerance to light and noise (migraine without aura)
- Neck pain accompanying a migraine attack

**Exclusion criteria**

- Neurological disease other than migraine
- Degenerative cervical condition
- Tension-type headache before the diagnosis of migraine
- History of vertigo
- History of hypertension
- Anxiety, depression, and sleep disturbances associated with a neurological or psychiatric disorder
- Fibromyalgia
- Cervicogenic headache
- History of cervical trauma
- Midtreatment absenteeism
- History of recent or previous systemic disease

### Interventions

The study will be conducted among male and female participants diagnosed with chronic migraine by a neurologist and selected based on specific inclusion and exclusion criteria. Participants will be enrolled through simple random sampling using computer-generated numbers. Study procedures will be explained in detail before obtaining informed consent. Using simple random sampling, participants will be randomly assigned to 2 groups: experimental group (t-SNS) and control group (ta-VNS). The experimental group (t-SNS) will receive supraorbital nerve stimulation and conventional physiotherapy, while the control group will receive ta-VNS and conventional physiotherapy. The allocation ratio is 1:1. The effectiveness of the assessment will be assessed by comparing preintervention and postintervention outcomes between groups. Follow-up assessments will be conducted immediately after the 4-week treatment period, at 8 weeks, and at 12 weeks. Nerve stimulation will be administered in 20-minute daily sessions for 4 weeks. Conventional physiotherapy will be administered 3 times per week for 4 weeks in both groups.

### Experimental Group: t-SNS (Group A)

t-SNS is also known as external trigeminal nerve stimulation. The maximum skin impedance of the constant-current generator is 2.2 kΩ. In clinical practice, electrical impulses are transmitted via a supraorbital electrode, which stimulates branches of the ophthalmic nerve (V1) located beneath the skin of the forehead. The parameters used for the electrical impulses will include a mean electric current of 0, an impulse width of 250 µs, a frequency of 60 Hz, and a maximum intensity of 16 mA, with a progressive slope from 1 to 16 mA. The electrode will be placed on the forehead. A transcutaneous electrical nerve stimulator device will be used for the stimulation.

The patient will receive 20-minute daily sessions for 4 weeks. As the current intensity progressively increases, the patient will be able to stabilize the intensity when the tingling or prickling sensation in the forehead becomes uncomfortable [13].

### Control Group: ta-VNS (Group B)

The vagal nerve stimulation (VNS) device will generate a proprietary electrical signal that provides a low voltage at its peak, 24 V, and a maximum output current of 60 mA [14]. The NEMOS ta-VNS stimulator device will be placed on the skin of the concha of the ear to provide sensory stimulation to the auricular branches of the vagus nerve. This handheld device will contain a stimulator with an ear electrode that will be placed on the skin of the concha. The device will automatically measure impedance; if the electrode is not in contact with the skin, an alarm will be triggered. During stimulation, a train of electrical pulses (pulse width 250 μs; frequency 1 Hz; duty cycle of 30 seconds on and 30 seconds off, to prevent habituation) will be applied to the skin of the concha. Stimulus intensity will be individually adjusted during visit 2 to elicit a tingling sensation without pain and may be further adjusted by participants as needed [15]. The patient will receive 20-minute daily sessions for 4 weeks.

### Conventional Physiotherapy (European Consensus Statement)

Conventional physiotherapy will be provided to both the experimental and control groups. It will include the following [16]:

- Warm-up (10 minutes of indoor cycling training)
- Exercise (30-45 minutes of walking, consisting of fast walking, an interval program [jogging and walking], and a continuous run of moderate intensity)
- Cool-down (5 minutes of breathing exercises and brisk walking)

This standardized protocol will be common to both groups.

### Follow-Up Period

Participants will receive a daily 20-minute stimulation session for 4 weeks, followed by 45 minutes of conventional physiotherapy after t-SNS or ta-VNS. The preintervention outcome measures will be recorded prior to the initiation of the intervention. Upon completion of the 4-week intervention, the postintervention outcome measures will be collected. To evaluate the long-term effects of the intervention, additional follow-up assessments will be conducted at the eighth week and 12th week after the intervention period.

### Outcomes

#### Primary Outcome: Reduction in Pain Intensity (Visual Analog Scale)

The Visual Analog Scale (VAS) will be used to assess pain intensity, whereby patients will rate their perceived pain intensity on a 10-cm horizontal line, where 0=“absence of pain” and 10=“worst pain imaginable.” It is considered a valid and reliable instrument, with an intraclass correlation coefficient (ICC) of 0.97 (95% CI 0.86-0.98) [17]. The primary outcome will be assessed at baseline, after 4 weeks of treatment, and at follow-up at 8 and 12 weeks.

#### Secondary Outcomes

##### Headache Days (Frequency)

The number of days on which participants experience a headache per month, the maximum pain intensity for each headache attack, and headache duration in hours per month will be recorded [18].

##### Migraine Frequency

Migraine frequency will be measured as the number of attacks per month or the number of days with migraine per month [3,19].

##### Headache Impact Test

The headache impact test–6 (HIT-6) measures the impact and effect of headache on the ability to function normally in daily life. The instrument consists of 6 questions, each with 5 verbal response categories. Finally, the total score will be obtained by summing the responses to all 6 items using the item weights specified. Scores ≥60 indicate severe life impact, scores of 56 to 59 indicate substantial life impact, scores of 50 to 55 indicate some life impact, and scores of ≤49 indicate little to no life impact. The reliability is 0.86, and the validity is 0.88 [20,21].

##### Migraine Disability Assessment Scale

The scale will be used to assess migraine-related disability in patients experiencing migraine. The Migraine Disability Assessment (MIDAS) score can be classified into grade I (0-5): minimal disability, grade II (6-10): mild incapacity, grade III (11-20): moderate disability, and grade IV (>21): severe disability [18]. It is a reliable measurement, with a Cronbach α of .83 and a Spearman correlation coefficient of 0.84 [22].

##### Neck Disability Index

The scale is used to assess disability due to neck pain, and the scoring is classified into 4 grades: mild (5-14 points), moderate (15-24 points), severe (25-34 points), or complete disability (≥35 points). This is the most recommended questionnaire for neck-related disability, with a Cronbach α of .74 and moderate reliability (ICC=0.50 or Spearman correlation=0.92) [22].

##### Cervical Range of Motion

The functionality of the cervical spine can be investigated by assessing active mobility. The cervical spine’s active range of motion (ARoM) is usually assessed in the sitting position and can be recorded in degrees of movement, such as flexion, extension, and rotation, by using a goniometer [23]. Passive range of motion (ROM) of the upper cervical segments (C1-C2) will also be assessed. The patients will then be assessed in a seated position with maximal flexion of the cervical spine, supported by the assessor. Passive rotation of the head to both sides was performed. ROM was measured with a goniometer. The movement will be arrested when the examiner feels a block sensation during the ROM or when the patient expresses pain. A ROM of <30°, compared with the reference value of 44° (ie, 14° less than the maximum), is considered a positive result. Furthermore, a difference of ≥10° between sides will also be considered a positive result [24].

### Sample Size

The sample size was calculated based on values from a reference article (Eslami et al [25]), considering the mean difference in change in headache intensity between baseline and after intervention. The mean change in headache intensity was 5.79 (SD 1.58) at baseline and 4.22 (SD 2.07) in the high-intensity aerobic exercise group (Textbox 2).

**Sample size calculation.**

**Parameters**

- Significance level (α)=.05; Z_1–α/2_=1.96
- β=.10; → Z_1–β_=1.28
- Mean in group 1 (μ_1_)=5.79
- SD in group 1 (σ_1_)=1.58
- Mean in group 2 (μ_2_)=4.377
- SD in group 2 (σ_2_)=2.07
- Allocation ratio (group 2/group 1; r)=1

**Noninferiority formula**

- n≥([Z_1–α/2_+Z_1–β_]^2^×[σ_1_^2^+σ_2_^2^/r])/(μ_1_–μ_2_)^2^

**Calculation**

Step 1: compute the difference in means:

- μ_1_–μ_2_=5.79–4.377=1.413

Step 2: compute variances:

- σ_1_^2^=(1.58)^2^=2.4964
- σ_2_^2^=(2.07)^2^=4.2849

Step 3: plug the values into the formula:

- n≥([1.96+1.28]^2^×[2.4964+4.2849])/(1.413)^2^

Step 4: calculate the numerator:

- 1.96+1.28=3.24
- (3.24)^2^=10.4976
- 2.4964+4.2849=6.7813
- Numerator=10.4976×6.7813=71.166

Step 5: calculate the denominator:

- (1.413)^2^=1.997

**Final calculation**

- n≥71.166/1.997≈35.65=36 per group

Considering a 10% dropout, the revised sample size will be 36+4=40 participants per group. Thus, the total sample size will be 40×2=80 participants.

### Recruitment

The estimated period for recruitment, intervention, and data collection will be approximately 3 months. Eligible participants attending the Physiotherapy OPD, Centre of Neurosciences and Medicine, Acharya Vinoba Bhave Rural Hospital, DMIHER (DU), Sawangi (Meghe), Wardha, will be recruited. The time required for participant recruitment and baseline assessment will be approximately 30 to 45 minutes.

### Randomization and Blinding

Participants will be allocated to either the experimental or control group by simple randomization. The process will follow a 1:1 allocation ratio. Allocation will be performed by the study coordinator, who will not be directly involved in data collection. The randomization list will be kept strictly confidential. Allocation will be performed by the study coordinator in a sequentially numbered fashion using identical, opaque, sealed envelopes.

The recruiting clinicians will be blinded to study group allocation and will not have access to the allocation sequence. Trained clinicians will enroll eligible participants. The study coordinator will independently assign participants to either the intervention or control group. Outcome assessors will be blinded. The analysis team will receive aggregated data for the control and experimental groups.

### Adverse Events and Assessment of Safety

After informed consent is obtained from participants and they are enrolled in the study, any adverse events will be collected and recorded throughout the study period. Any serious adverse event that occurs will be reported to the principal investigator and documented.

### Data Management and Monitoring

Data will be entered onto a paper-based clinical research form, which will be designed by the research team, by trained clinicians. Data for each participant will be entered, once data collection has been completed, into an electronic database containing checks to ensure completeness of data. Throughout the study, data monitoring will take place on a periodic basis. The database, once verified, will be appropriately saved. The database, once verified to be accurate, will be locked and submitted to an independent statistical team for analysis.

### Statistical Analysis

Stata (version 16.0; StataCorp) was used to perform statistical analyses. The primary analysis will follow the intention-to-treat (ITT) principle, including all randomized participants in their allocated intervention groups regardless of protocol deviations or treatment adherence. A per-protocol (PP) analysis will also be performed as a sensitivity analysis, including only participants who complete the study according to the protocol. The primary and secondary outcomes will be analyzed using a mixed-design repeated-measures ANOVA (mixed ANOVA), with group (intervention A and intervention B) as the between-subject factor and time (baseline, after intervention, and follow-up, where applicable) as the within-subject factor. The group×time interaction will be considered the primary indicator of treatment effectiveness. When significant interaction effects are observed, Bonferroni-adjusted post hoc comparisons will be conducted to identify differences between groups and time points. If baseline imbalances are identified or adjustment is required, analysis of covariance (ANCOVA) will be performed using baseline values as covariates to compare postintervention outcomes. For data that do not satisfy parametric assumptions, the Wilcoxon signed-rank test will be used for within-group comparisons, and the Mann-Whitney *U* test will be used for between-group comparisons. Effect sizes will be reported as partial η² for ANOVA or ANCOVA and Cohen *d* for between-group comparisons.

Missing data will be assessed to determine the pattern and mechanism of missingness using Little’s missing completely at random (MCAR) test. If the proportion of missing data is less than 5%, a complete-case analysis will be performed. If missing data exceed 5%, multiple imputation (MI) using chained equations will be applied under the assumption that the data are missing at random (MAR), and the primary ITT analysis will be conducted using the imputed dataset. Baseline values of the primary and secondary outcome measures will be included as covariates where appropriate to improve statistical precision and account for any residual baseline imbalance between groups. To control the risk of type I error arising from multiple comparisons, 1 predefined primary outcome will be tested at a significance level of α=.05, while Bonferroni correction will be applied for multiple secondary outcomes and post hoc analyses. All adverse events occurring during the intervention period will be recorded and classified according to severity and their relationship to the intervention. The incidence of adverse events will be summarized using frequencies and percentages and compared between groups using the chi-square test or Fisher exact test, as appropriate. Sensitivity analyses will be performed by comparing the results of the ITT and PP analyses, as well as the findings obtained from complete-case and MI datasets. All statistical tests will use a significance threshold of α=.05 and will be 2-sided. At *P*<.05, differences will be deemed statistically significant.

### Ethical Considerations

The study was registered with the Clinical Trials Registry–India (CTRI) on January 1, 2026, and was approved by the DMIHER (DU) Institutional Ethics Committee (DMIHER[DU]/IEC/2025/562) on October 3, 2025. Prior to their involvement in the study, each participant will provide written informed consent. There is no danger or damage associated with withdrawing from the research at any time, and participation is completely optional. The principal investigator will permit participants to withdraw from the study at any time without imposing any penalties. All information gathered for the study will be kept completely private and used only for research; participant names will not be revealed through disclosure or publication.

## Results

The CTRI has registered the research protocol, which was approved by the institutional ethics committee. The study was initiated after obtaining ethics approval. No funding has been received from the institution. Recruitment is ongoing and will end in July 2027. As of the time of manuscript preparation, 5 patients have been recruited. The first participant was enrolled on March 8, 2026. Upon completion of data collection, statistical analysis will be performed by a qualified statistician, and the findings are expected to be published in 2027.

## Discussion

### Anticipated Findings

Over time, the pharmaceutical treatment of migraine has changed, shifting from a mostly symptomatic approach to a more focused one. For both acute and preventive care, triptans, nonsteroidal anti-inflammatory drugs (NSAIDs), and oral preventive drugs continue to be important choices [26]. Compared to opioids, ergotamine and triptan-containing medications were shown to be more advantageous in a comprehensive review of 29 studies. However, there is a 2-fold increase in migraine development associated with opiates and barbiturates. Conversely, those with a high baseline frequency of migraines are more likely to develop chronic headaches when using triptans. Anti-inflammatory medications, such as NSAIDs, were protective in those who had a lower frequency of headache (<10 days), but similar to triptans, they were associated with an increased risk of migraine progression in those who had a high frequency of headache at baseline (>10 days) [27]. Even with the appropriate use of current acute treatments, there are still unmet therapeutic needs, as not all patients may benefit from these available treatments [27]. Drug therapy, both preventive and abortive, is the foundation of most therapeutic approaches. Conventional pharmaceutical treatments, however, have severe side effects and are only partially successful. Drug resistance and possibly the development of refractory medication-overuse headaches can result from overuse of symptomatic headache medications. Therefore, nonpharmacological therapeutic strategies with better efficacy and tolerance are urgently needed [28]. ta-VNS and t-SNS represent noninvasive neuromodulation techniques that act directly on central pain-processing pathways implicated in migraine pathophysiology. Both modalities influence key brain regions involved in nociceptive modulation, including the trigeminovascular system, locus coeruleus, nucleus tractus solitarius, and cortical pain networks. Previous evidence suggests that ta-VNS enhances parasympathetic activity and activates descending inhibitory pathways, thereby reducing cortical hyperexcitability and central sensitization. Similarly, t-SNS modulates trigeminal nerve afferents, leading to decreased cortical excitability and reduced propagation of migraine-related nociceptive signals [29].

Several studies have demonstrated that both ta-VNS and t-SNS can significantly reduce migraine attack frequency, intensity, and duration when compared with conventional physiotherapy or sham interventions alone. These neuromodulatory effects appear to be more consistent and sustained because of direct central nervous system engagement rather than solely peripheral musculoskeletal mechanisms. Although conventional physiotherapy contributes to symptom relief through postural correction, muscle relaxation, and improved cervical biomechanics, it may not adequately address central sensitization, which plays a critical role in chronic migraine.

The incorporation of ta-VNS and t-SNS alongside conventional physiotherapy may therefore provide a multimodal approach, targeting both central and peripheral mechanisms of migraine. However, limited comparative evidence exists regarding the relative efficacy of ta-VNS vs t-SNS when combined with conventional physiotherapy. This study aims to address this gap by evaluating and comparing their effects on pain intensity, migraine frequency, and functional outcomes. The findings may contribute to evidence-based clinical decision-making and help optimize nonpharmacological management strategies for migraine.

### Conclusions

This study protocol proposes to evaluate and compare the effectiveness of ta-VNS and t-SNS combined with conventional physiotherapy in the management of migraine. By targeting central pain modulation pathways along with peripheral musculoskeletal components, the proposed interventions aim to address key mechanisms involved in migraine pathophysiology. The findings of this study are expected to provide valuable evidence regarding the comparative efficacy of these noninvasive neuromodulation techniques and may help to identify an effective, safe, and clinically feasible adjunct to conventional physiotherapy for migraine management. The results may also contribute to the development of standardized, evidence-based physiotherapy protocols for migraine care.

## Appendix

Multimedia Appendix 1 SPIRIT Checklist.

## Abbreviations

ANCOVA: analysis of covariance
ARoM: active range of motion
CSD: cortical spreading depression
CTRI: Clinical Trials Registry–India
DMIHER: Datta Meghe Institute of Higher Education and Research
DU: deemed to be university
GBD: Global Burden of Disease
HIT-6: headache impact test–6
ICC: intraclass correlation coefficient
ICD-10: International Classification of Diseases, 10th Revision
ICD-9: International Classification of Diseases, 9th Revision
ICHD-3: International Classification of Headache Disorders–3
ITT: intention-to-treat
MAR: missing at random
MCAR: missing completely at random
MI: multiple imputation
MIDAS: Migraine Disability Assessment
NSAID: nonsteroidal anti-inflammatory drug
OPD: outpatient department
PP: per-protocol
ROM: range of motion
ta-VNS: transcutaneous auricular vagal nerve stimulation
t-SNS: transcutaneous supraorbital nerve stimulation
VAS: visual analog scale
VNS: vagal nerve stimulation
WHO: World Health Organization
YLD: year lived with disability

## References

[1] Ravisankar P, Hundia A, Sindhura J, Rani BS, Anvith PS, Pragna P (2015). Migraine - a comprehensive review. Indo Am J Pharm Res.

[2] Dong H, Dong W, Jin Y, Jiang Y, Li Z, Yu D (2025). The global burden of migraine: a 30-year trend review and future projections by age, sex, country, and region. Pain Ther.

[3] Eigenbrodt AK, Ashina H, Khan S, Diener HC, Mitsikostas DD, Sinclair AJ, Pozo-Rosich P, Martelletti P, Ducros A, Lantéri-Minet M, Braschinsky M, Del Rio MS, Daniel O, Özge A, Mammadbayli A, Arons M, Skorobogatykh K, Romanenko V, Terwindt GM, Paemeleire K, Sacco S, Reuter U, Lampl C, Schytz HW, Katsarava Z, Steiner TJ, Ashina M (2021). Diagnosis and management of migraine in ten steps. Nat Rev Neurol.

[4] Chhater S, Karal R, Kumar B (2018). Review on migraine: pathophysiology and treatment. Am J Biomed Res.

[5] Vicente BN, Oliveira R, Martins IP, Gil-Gouveia R (2023). Cranial autonomic symptoms and neck pain in differential diagnosis of migraine. Diagnostics (Basel).

[6] Onan D, Ekizoğlu E, Arıkan H, Taşdelen B, Özge A, Martelletti P (2023). The efficacy of physical therapy and rehabilitation approaches in chronic migraine: a systematic review and meta-analysis. J Integr Neurosci.

[7] Kim KM, Kim BK, Lee W, Hwang H, Heo K, Chu MK (2022). Prevalence and impact of visual aura in migraine and probable migraine: a population study. Sci Rep.

[8] del Rio MS, Cutrer FM (2023). Chapter 6 - Pathophysiology of migraine aura. Handbook of Clinical Neurology.

[9] Chowdhury D, Krishnan A, Duggal A, Amarchand R, Husøy A, Steiner TJ (2024). Headache prevalence and demographic associations in the Delhi and National Capital Region of India: estimates from a cross-sectional nationwide population-based study. J Headache Pain.

[10] Dabi RD, Rajmanyal AV, Chaudhari CR, Chaudhari TS (2025). Cross-sectional study on the prevalence of migraine in working adults. Neuroquantology.

[11] Al-Khazali HM, Younis S, Al-Sayegh Z, Ashina S, Ashina M, Schytz HW (2022). Prevalence of neck pain in migraine: a systematic review and meta-analysis. Cephalalgia.

[12] Steiner TJ, Stovner LJ, Jensen R, Uluduz D, Katsarava Z, Lifting The Burden: the Global Campaign against Headache (2020). Migraine remains second among the world's causes of disability, and first among young women: findings from GBD2019. J Headache Pain.

[13] Riederer F, Penning S, Schoenen J (2015). Transcutaneous supraorbital nerve stimulation (t-SNS) with the Cefaly® device for migraine prevention: a review of the available data. Pain Ther.

[14] Silberstein SD, Calhoun AH, Lipton RB, Grosberg BM, Cady RK, Dorlas S, Simmons KA, Mullin C, Liebler EJ, Goadsby PJ, Saper JR (2016). Chronic migraine headache prevention with noninvasive vagus nerve stimulation: the EVENT study. Neurology.

[15] Straube A, Ellrich J, Eren O, Blum B, Ruscheweyh R (2015). Treatment of chronic migraine with transcutaneous stimulation of the auricular branch of the vagal nerve (auricular t-VNS): a randomized, monocentric clinical trial. J Headache Pain.

[16] Lemmens J, De Pauw J, Van Soom T, Michiels S, Versijpt J, van Breda E, Castien R, De Hertogh W (2019). The effect of aerobic exercise on the number of migraine days, duration and pain intensity in migraine: a systematic literature review and meta-analysis. J Headache Pain.

[17] Muñoz-Gómez E, Inglés M, Aguilar-Rodríguez M, Mollà-Casanova S, Sempere-Rubio N, Serra-Añó P, Espí-López GV (2022). Effect of a craniosacral therapy protocol in people with migraine: a randomized controlled trial. J Clin Med.

[18] Wanderley D, Valença MM, de Souza Costa Neto JJ, Martins JV, Raposo MC, de Oliveira DA (2020). Contract-relax technique compared to static stretching in treating migraine in women: a randomized pilot trial. J Bodyw Mov Ther.

[19] La Touche R, Fierro-Marrero J, Sánchez-Ruíz I, Rodríguez de Rivera-Romero B, Cabrera-López CD, Lerma-Lara S, Requejo-Salinas N, de Asís-Fernández F, Elizagaray-García I, Fernández-Carnero J, Matesanz-García L, Pardo-Montero J, Paris-Alemany A, Reina-Varona Á (2023). Prescription of therapeutic exercise in migraine, an evidence-based clinical practice guideline. J Headache Pain.

[20] Torlak MS, Atıcı E, Cıbık M (2022). Effects of transcutaneous occipital nerve stimulation and instrument-assisted soft tissue mobilization in chronic migraine. J Manip Physiol Ther.

[21] Houts CR, McGinley JS, Wirth RJ, Cady R, Lipton RB (2021). Reliability and validity of the 6-item Headache Impact Test in chronic migraine from the PROMISE-2 study. Qual Life Res.

[22] Bevilaqua-Grossi D, Gonçalves MC, Carvalho G, Florencio L, Dach F, Speciali J, Bigal ME, Chaves TC (2016). Additional effects of a physical therapy protocol on headache frequency, pressure pain threshold, and improvement perception in patients with migraine and associated neck pain: a randomized controlled trial. Arch Phys Med Rehabil.

[23] Di Antonio S, Arendt-Nielsen L, Castaldo M (2023). Cervical musculoskeletal impairments and pain sensitivity in migraine patients. Musculoskelet Sci Pract.

[24] Carvalho GF, Lodovichi SS, Pinheiro CF, Benatto MT, Florencio LL, Bragatto MM, Dach F, Bevilaqua-Grossi D (2021). The presence of aura is not related to changes in the cervical performance and mobility of patients with migraine. Musculoskelet Sci Pract.

[25] Eslami R, Parnow A, Pairo Z, Nikolaidis P, Knechtle B (2021). The effects of two different intensities of aerobic training protocols on pain and serum neuro-biomarkers in women migraineurs: a randomized controlled trail. Eur J Appl Physiol.

[26] Pellesi L, Do TP, Hougaard A (2024). Pharmacological management of migraine: current strategies and future directions. Expert Opin Pharmacother.

[27] Ong JJ, De Felice M (2018). Migraine treatment: current acute medications and their potential mechanisms of action. Neurotherapeutics.

[28] Tao H, Wang T, Dong X, Guo Q, Xu H, Wan Q (2018). Effectiveness of transcutaneous electrical nerve stimulation for the treatment of migraine: a meta-analysis of randomized controlled trials. J Headache Pain.

[29] Naghdi S, Underwood M, Madan J, Brown A, Duncan C, Matharu M, Aksentyte A, Davies N, Rees S, Cooklin A, Grove A, Mistry H (2023). Clinical effectiveness of pharmacological interventions for managing chronic migraine in adults: a systematic review and network meta-analysis. J Headache Pain.

